# Contraception Use and Pregnancy Risk Among Adolescents in Pediatric Emergency Departments

**DOI:** 10.1001/jamanetworkopen.2024.18213

**Published:** 2024-06-28

**Authors:** Hannah Canter, Jennifer Reed, Chella Palmer, T. Charles Casper, Kristin Stukus, Sarah Schmidt, Michelle Pickett, Cynthia Mollen, Cara Elsholz, Andrea T. Cruz, Erin Augustine, Monika K. Goyal

**Affiliations:** 1 Department of Pediatrics, Oregon Health and Science University, Portland; 2 Division of Emergency Medicine, Cincinnati Children’s Hospital Medical Center, Department of Pediatrics, University of Cincinnati College of Medicine, Cincinnati, Ohio; 3 Department of Pediatrics, University of Utah, Salt Lake City; 4 Department of Pediatrics, Nationwide Children’s Hospital, Ohio State University, Columbus; 5 Department of Pediatrics, Children’s Hospital Colorado, University of Colorado School of Medicine, Aurora; 6 Department of Pediatrics, Medical College of Wisconsin, Milwaukee; 7 Department of Pediatrics, Perelman School of Medicine at the University of Pennsylvania, Philadelphia; 8 Department of Pediatrics, Baylor College of Medicine, Houston, Texas; 9 Department of Pediatrics, Ann & Robert H. Lurie Children’s Hospital, Northwestern University Feinberg School of Medicine, Chicago, Illinois; 10 Department of Pediatrics, Children’s National Hospital, George Washington University, Washington, DC

## Abstract

**Importance:**

Unintended pregnancy is a major health risk for adolescents in the US, and adolescents face many barriers to obtaining effective and reliable contraception.

**Objective:**

To measure and describe the use of contraception, pregnancy risk index (PRI), and emergency contraception (EC) prescriptions among female adolescents accessing the emergency department (ED) for care.

**Design, Setting, and Participants:**

This cross-sectional study is a planned secondary analysis of a multicenter trial from April 2021 through April 2022 that used a tablet-based, content-validated, confidential sexual health survey at 6 urban, pediatric tertiary care EDs affiliated with the Pediatric Emergency Care Applied Research Network. Participants were individuals aged 15 to 21 years presenting to the ED who completed the confidential sexual health survey and indicated female sex assigned at birth and prior penile-vaginal sexual intercourse. Data analysis was performed from January 2023 to February 2024.

**Main Outcomes and Measures:**

The primary outcomes were the type and proportion of contraception use, the PRI, and provision of EC. Separate multivariable logistic regression models were performed to identify sociodemographic factors associated with these outcomes.

**Results:**

A total of 1063 participants (median [IQR] age, 17.5 [16.5-18.3] years) were included in this analysis; 219 (20.8%) identified as Hispanic, 464 (44.1%) identified as non-Hispanic Black, 308 (29.3%) identified as non-Hispanic White, and 61 (5.8%) identified as other races and ethnicities. In total, 756 participants (71.1%) reported contraception use during their last sexual encounter. Long-acting reversible contraception use (LARC) was the least used (164 participants [15.4%]), and 307 (28.9%) reported no contraception use. Sociodemographic factors associated with overall contraception use, and LARC use specifically, included insurance and race and ethnicity. The overall PRI was 7.89, or an expected 8 pregnancies per 100 female individuals per year. Although 108 participants (10.2%) were eligible for EC, EC was ordered for only 6 (5.6%) of those eligible.

**Conclusions and Relevance:**

In this cross-sectional study of sexually active adolescents presenting to the ED, the majority of participants reported using at least 1 form of contraception; however, LARCs were the least used option, and 28.9% of participants reported no contraceptive use. The unintended pregnancy risk was almost 8% in the study population. Few patients eligible for EC received it. These data suggest a high need and potential opportunity for provision of contraception services in the ED setting.

## Key Points

**Question:**

Among adolescents seeking emergency department (ED) care, what is the prevalence of contraceptive use and pregnancy risk, and what proportion of those eligible receive emergency contraception (EC)?

**Findings:**

In this cross-sectional study of 1063 individuals seen at 6 pediatric EDs, 28.9% of sexually active female adolescents reported no contraception use, yielding a pregnancy risk index of 7.89 expected pregnancies per 100 adolescents annually. Although 10.2% of participants were eligible for EC, only 5.6% of those eligible received it.

**Meaning:**

In this study, adolescents seeking ED care were at high risk for pregnancy and there is an opportunity to expand pregnancy prevention services in the ED setting.

## Teaser

This cross-sectional study examines the use of contraception, pregnancy risk index, and emergency contraception prescriptions among female adolescents in pediatric emergency departments (EDs).

## Introduction

Unintended pregnancy is a major public health problem that affects thousands of adolescents in the US each year. Despite a general decline in US teen pregnancy rates over the last several decades, they remain the highest among developed countries.^1^ In 2019, the pregnancy rate among female adolescents aged 15 to 19 years was 29.4 per 1000 individuals.^2^ Furthermore, the vast majority (70.8%) of these pregnancies were unintended.^2^ In one study^3^ conducted in an urban pediatric emergency department (ED), most female adolescents aged 15 to 19 years reported they wanted to avoid pregnancy, yet one-third were either pregnant or at high risk of becoming pregnant in the next year. This indicates a substantial unmet need for effective and reliable contraception among teenagers.

There are many barriers to contraception access for adolescents. Policies on minors’ ability to consent for contraceptive care vary from state to state.^4^ Even in states where adolescents can legally access services, other barriers such as cost and lack of transportation, education, and confidentiality remain.^5^ Many adolescents do not have an established primary care home and, therefore, use the ED as their primary source of health care.^6^ Multiple studies^3,7^ have demonstrated suboptimal contraceptive use and high rates of unintended pregnancy risk in the adolescent ED population.

Thus, we conducted this secondary analysis of a multicenter study to measure and describe the use of contraception and pregnancy risk among adolescents accessing the ED for care. We also sought to identify provision of emergency contraception (EC) to adolescents who were eligible for it on the basis of their sexual behaviors and contraception use.

## Methods

### Study Design

This cross-sectional study is a secondary analysis of a multicenter trial that used tablet-based, patient-entered data to trigger electronic health record–embedded clinical decision support for broad-scale sexually transmitted infection screening at 6 urban, pediatric tertiary care EDs across the Pediatric Emergency Care Applied Research Network from April 1, 2021, through April 30, 2022. This study was approved by each institution’s institutional review board through a single institutional review board mechanism with a waiver of parental consent for participation. All study participants provided informed consent via a tablet computer before participation. This study follows the Strengthening the Reporting of Observational Studies in Epidemiology (STROBE) reporting guidelines for cross-sectional studies.

### Study Population

The study used a content-validated computerized sexual health survey (cSHS).^8^ The survey was provided in English. Patients were eligible to complete the survey if they were aged 15 to 21 years and had no cognitive or developmental delay, critical illness, or altered mental status that would prohibit them from completing the cSHS. Patients were included in this secondary analysis if they identified sex assigned at birth as female, indicated they had engaged in penile-vaginal sex, and answered the contraception use question on the cSHS. Race and ethnicity options were defined and identified by the electronic health record (Hispanic or Latino, non-Hispanic Black, non-Hispanic White, and other, which includes American Indian or Alaska Native, Asian or Pacific Islander, and any other race or ethnicity not otherwise specified).

### Outcomes

The primary outcomes included use and type of contraception during the last sexual encounter. These data were then used to calculate the pregnancy risk index (PRI), a validated measure that summarizes pregnancy risk among all adolescents (including those identified as non–sexually active).^9^ A secondary outcome was prescription of EC during the ED visit for those who were eligible.

### Statistical Analysis

We used descriptive statistics to summarize patient characteristics, contraception use and type, and EC provision using frequencies and percentages. Because participants could indicate multiple contraception methods, we also identified highest-ranking contraception use as determined by lowest failure rate, with failure rate defined as the percentage of participants experiencing pregnancy in the first year of use. The ranking of contraception methods from lowest to highest failure rate is as follows: contraceptive implant (0.1%), intrauterine device (IUD) (0.6%), injectable (4.0%), combined hormonal methods (pill, patch, and vaginal ring) (7.0%), and male condom (13.0%).^10^

We performed multivariable logistic regression to identify sociodemographic factors associated with any contraception use. Because long-acting reversible contraception (LARC) is considered the most effective form of contraception and is recommended by the American Academy of Pediatrics as the first-line contraceptive choice for adolescents,^10^ we developed a separate multivariable model to identify factors associated with LARC use. LARC use was defined as the use of an IUD or implant. Covariables included in the models were age, race and ethnicity, insurance status, timing of most recent sexual encounter, and number of sexual partners in the previous 3 months. Race and ethnicity were included as covariables in the multivariable models because they are social constructs that serve as markers of structural racism and its impact on access to quality health care. The initial analysis also included a comparison between abortion-protected and abortion-not-protected sites, as defined by the Center for Reproductive Rights; however, owing to the rapidly changing landscape of abortion laws, this was not included in the final analysis.^11^

PRI was calculated by site and overall. The PRI summarizes the risk of pregnancy among all adolescents. A PRI of 5, for example, equates to 5 expected pregnancies per 100 female adolescents per year. The PRI is calculated as the product of 2 components: the percentage of the population currently sexually active and the contraception risk index (CRI). To calculate the PRI, we first calculated the CRI, a validated composite measure using contraceptive use data combined with contraceptive failure rates.^9,12^ This represents pregnancy risk for the sexually active proportion of that population by summing the product of each method’s specific failure rate and the proportion of those who are sexually active using that method at their most recent sexual intercourse. In these calculations, the nonuse of contraception is included with a specific risk of pregnancy. If a patient indicated multiple methods, then the method with the lowest contraceptive failure rate was used for the calculation. The CRI is multiplied by the percentage of the population that is currently sexually active to determine the PRI. For our analysis, we considered the percentage of the population that is currently sexually active based on the percentage of female adolescents in our sample who were eligible for this analysis of all female adolescents in the sample. We also evaluated EC ordering for eligible participants. Participants were considered eligible if they reported most recent sexual intercourse less than 5 days ago and highest-ranking form of contraception reported as no method or withdrawal.

Adjusted odds ratios (aORs) and 95% CIs were calculated, with significance defined as a 95% CI that did not include the reference value of 1. Analyses were performed using SAS statistical software version 9.4 (SAS Institute). Data analysis was performed from January 2023 to February 2024.

## Results

A total of 5136 adolescents completed the cSHS between April 1, 2021, and April 30, 2022; 3138 (61.1%) identified as female sex at birth, of whom 1171 (37.3%) reported engaging in penile-vaginal sexual intercourse. Of these patients, 1063 (90.8%) completed the survey item related to contraception use and compose the study population for this secondary analysis. These patients had a median (IQR) age of 17.5 (16.5-18.3) years and identified as Hispanic (219 patients [20.8%]), non-Hispanic Black (464 patients [44.1%]), non-Hispanic White (308 patients [29.3%]), and other races and ethnicities (61 patients [5.8%]). The majority (693 patients [65.2%]) had government insurance at the time of visit (Table 1).

**Table 1.** Study Population by Contraception Use

Characteristic	Participants, No. (%)		
	Overall (N = 1063)	Used contraceptives (n = 756)	Did not use contraceptives (n = 307)
Age at arrival date, median (IQR), y	17.5 (16.5-18.3)	17.5 (16.5-18.2)	17.5 (16.5-18.4)
Race and ethnicity			
Hispanic or Latino	219 (20.8)	163 (21.8)	56 (18.4)
Non-Hispanic Black	464 (44.1)	283 (37.8)	181 (59.5)
Non-Hispanic White	308 (29.3)	253 (33.8)	55 (18.1)
Other^a^	61 (5.8)	49 (6.6)	12 (3.9)
Insurance			
Government	693 (65.2)	447 (59.1)	246 (80.1)
Commercial	347 (32.6)	293 (38.8)	54 (17.6)
Other	23 (2.2)	16 (2.1)	7 (2.3)
Current gender identity			
Female	1020 (96.8)	725 (96.8)	295 (96.7)
Male	9 (0.9)	6 (0.8)	3 (1.0)
Gender queer or gender nonconforming	18 (1.7)	13 (1.7)	5 (1.6)
Other	7 (0.7)	5 (0.7)	2 (0.7)
Last sexual intercourse			
≤5 d	326 (32.7)	211 (29.7)	115 (40.1)
>5 d to <1 mo	315 (31.6)	234 (33.0)	81 (28.2)
>1 mo to <1 y	282 (28.3)	208 (29.3)	74 (25.8)
>1 y	74 (7.4)	57 (8.0)	17 (5.9)
No. of sexual partners in last 3 mo			
0	139 (13.2)	106 (14.1)	33 (10.9)
1	772 (73.2)	558 (74.4)	214 (70.4)
≥2	143 (13.6)	86 (11.5)	57 (18.8)
Study site			
A	436 (41.0)	302 (39.9)	134 (43.6)
B	182 (17.1)	142 (18.8)	40 (13.0)
C	46 (4.3)	29 (3.8)	17 (5.5)
D	107 (10.1)	80 (10.6)	27 (8.8)
E	264 (24.8)	189 (25.0)	75 (24.4)
F	28 (2.6)	14 (1.9)	14 (4.6)
Sexual contact			
Penis in vagina sexual contact	1063 (100.0)	756 (100.0)	307 (100.0)
Vagina on vagina sexual contact	73 (6.9)	48 (6.3)	25 (8.1)
Anal sexual contact	116 (10.9)	80 (10.6)	36 (11.7)
Oral sexual contact	557 (52.4)	399 (52.8)	158 (51.5)

^a^ Other race and ethnicity includes American Indian or Alaska Native, Asian or Pacific Islander, and any other race or ethnicity.

### Contraception Use

Among the study cohort, 756 participants (71.1%) reported some form of contraception use in their most recent sexual encounter, 265 (24.9%) reported use of multiple concurrent methods, and 307 (28.9%) reported no contraception use. When sorted by highest-ranking method, 312 (29.4%) used a short-acting hormonal method (injectable, patch, pill, or vaginal ring), 260 (24.5%) used male condoms, 164 (15.4%) used LARC (implant or IUD), 82 (7.7%) used withdrawal, and 50 (4.7%) used EC, whereas 195 (18.3%) used no method (Table 2). Thus, of the 756 participants who were using contraception, 494 (65.3%) reported using a highly effective method, defined as any hormonal method or IUD.

**Table 2.** Contraception Use Methods During Last Sexual Intercourse

Method	Participants, No. (%)	
	Overall (N = 1063)^a^	Highest ranking contraception used by patient^b^
Implant	95 (8.9)	95 (8.9)
Intrauterine device	69 (6.5)	69 (6.5)
Emergency contraception	50 (4.7)	50 (4.7)
Injectable	103 (9.7)	102 (9.6)
Pill	202 (19.0)	189 (17.8)
Vaginal ring	12 (1.1)	8 (0.8)
Patch	13 (1.2)	13 (1.2)
Male condom	454 (42.7)	260 (24.5)
Withdrawal	196 (18.4)	82 (7.7)
No method	207 (19.5)	195 (18.3)

^a^ The overall column is not mutually exclusive, and participants can be counted in multiple contraceptive methods.

^b^ The highest ranking contraception column is mutually exclusive, and highest ranking is determined by contraception failure rates.

Multivariable models showed significant differences in contraception use by several different patient characteristics (Table 3). The following aORs were based on preventive contraception use and do not include EC or withdrawal. With regard to race and ethnicity, participants identifying as non-Hispanic White (aOR, 2.14; 95% CI, 1.44-3.20), Hispanic (aOR, 1.90; 95% CI, 1.31-2.78), and other race and ethnicity (aOR, 2.25; 95% CI, 1.17-4.64) had higher odds of contraception use than non-Hispanic Black participants. Participants with commercial insurance (aOR, 2.58; 95% CI, 1.78-3.80) were more likely to use contraception than those with government insurance. Participants reporting their last sexual intercourse between 5 days and 1 month ago (aOR, 1.68; 95% CI, 1.17-2.41) and those between 1 month and 1 year ago (aOR, 1.61; 95% CI, 1.08-2.40) had higher odds of contraception use than those with more recent (within the past 5 days) sexual encounters. The number of sexual partners in the last 3 months was not significantly associated with the odds of contraception use.

**Table 3.** Sociodemographic Factors Associated With Contraception Use^a^

Factor	Contraception use, aOR (95% CI)	
	Univariable model	Multivariable model^b^
Age at arrival date, y		
15 to <18	1 [Reference]^c^	1 [Reference]^c^
18 to <22	0.76 (0.57-1.01)	0.95 (0.70-1.30)
Race and ethnicity		
Hispanic or Latino	1.86 (1.31-2.67)	1.90 (1.31-2.78)
Non-Hispanic Black	1 [Reference]^c^	1 [Reference]^c^
Non-Hispanic White	2.94 (2.09-4.19)	2.14 (1.44-3.20)
Other^d^	2.61 (1.40-5.27)	2.25 (1.17-4.64)
Insurance at visit		
Government	1 [Reference]^c^	1 [Reference]^c^
Commercial	2.99 (2.16-4.18)	2.58 (1.78-3.80)
Other	1.26 (0.53-3.31)	1.10 (0.43-3.04)
Last sexual intercourse		
≤5 d	1 [Reference]^c^	1 [Reference]^c^
>5 d to <1 mo	1.57 (1.12-2.22)	1.68 (1.17-2.41)
>1 mo to <1 y	1.53 (1.08-2.18)	1.61 (1.08-2.40)
>1 y	1.83 (1.04-3.37)	1.63 (0.75-3.61)
No. of sexual partners in the last 3 mo		
0	1 [Reference]^c^	1 [Reference]^c^
1	0.81 (0.53-1.22)	1.10 (0.60-1.97)
≥2	0.47 (0.28-0.78)	0.58 (0.29-1.15)

Abbreviation: aOR, adjusted odds ratio.

^a^ Contraception methods do not include emergency contraception or withdrawal method.

^b^ Adjusted for the variables that were statistically significant in the univariable model.

^c^ Reference categories are based on the most frequent variable.

^d^ Other race and ethnicity includes American Indian or Alaska Native, Asian or Pacific Islander, and any other race or ethnicity.

### LARC Use

A total of 164 participants (15.4%) reported LARC use. Multivariable models of the association of patient characteristics with LARC are shown in Table 4. Similar to differences in general contraceptive by race and ethnicity, study participants who identified as non-Hispanic White (aOR, 2.81; 95% CI, 1.77-4.50), Hispanic (aOR, 1.87; 95% CI, 1.14-3.06), and other races and ethnicities (aOR, 2.36; 95% CI, 1.11-4.46) had higher odds of LARC use than non-Hispanic Black participants. Regarding timing of most recent sexual encounter, participants reporting their most recent sexual intercourse between 1 month and 1 year ago had lower odds of LARC use than those with last sexual encounter within last 5 days (aOR, 0.47; 95% CI, 0.28-0.77).

**Table 4.** Sociodemographic Factors Associated With LARC Use^a^

Variable	LARC use, aOR (95% CI)	
	Univariable model	Multivariable model^b^
Age at arrival date, y		
15 to <18	1 [Reference]	NA
18 to <22	1.08 (0.75-1.53)	NA
Race and ethnicity		
Hispanic or Latino	2.04 (1.27-3.28)	1.87 (1.14-3.06)
Non-Hispanic Black	1 [Reference]	1 [Reference]
Non-Hispanic White	2.90 (1.92-4.42)	2.81 (1.77-4.50)
Other^c^	2.72 (1.32-5.31)	2.36 (1.11-4.76)
Insurance at visit		
Government	1 [Reference]	1 [Reference]
Commercial	1.52 (1.08-2.15)	1.12 (0.75-1.67)
Other	0.96 (0.22-2.86)	0.59 (0.09-2.12)
Last sexual intercourse		
≤5 d	1 [Reference]	1 [Reference]
>5 d to <1 mo	0.67 (0.45-1.01)	0.65 (0.42-0.98)
>1 mo to <1 y	0.45 (0.28-0.71)	0.47 (0.28-0.77)
>1 y	0.51 (0.23-1.02)	0.55 (0.19-1.47)
No. sexual partners in the last 3 mo		
0	1 [Reference]	1 [Reference]
1	1.82 (1.05-3.41)	1.60 (0.73-3.72)
≥2	1.20 (0.57-2.58)	1.01 (0.39-2.68)

Abbreviations: aOR, adjusted odds ratio; LARC, long-acting reversible contraception; NA, not applicable.

^a^ LARC includes intrauterine devices and implants.

^b^ Adjusted for the variables that were statistically significant in the univariable model.

^c^ Other race and ethnicity includes American Indian or Alaska Native, Asian or Pacific Islander, and any other race or ethnicity.

### Pregnancy Risk Index

Calculation of the PRI was based on all adolescents assigned female sex at birth who completed the cSHS during the same study period, regardless of reported sexual activity. Of these 3138 patients, 3030 (96.6%) completed the survey item related to contraception use and were included in the PRI calculation. The overall PRI for the study population was 7.89, or an expected 8 pregnancies per 100 female adolescents per year. The PRIs calculated for each site were as follows: site A, 8.41; site B, 8.31; site C, 6.47; site D, 8.41; site E, 7.05; and site F, 7.83.

### EC Administration

A total of 108 study participants (10.2%) were considered potentially eligible for EC (based on report of most recent sexual intercourse <5 days ago and highest-ranking form of contraception reported as no method or withdrawal). Among this group, EC was ordered for 6 total participants, or 5.6%. There were no significant differences between withdrawal and reporting no method on provision of EC.

## Discussion

This cross-sectional study demonstrates that, although most (71.1%) sexually active adolescents presenting to the ED were using at least 1 form of contraception, 28.9% reported no contraceptive use in their most recent sexual encounter. The recent Youth Behavior Surveillance System survey and the National Survey of Family Growth reported national rates of female adolescents not using contraception in their most recent sexual encounters as 13.8% and 9.8%, respectively.^13,14^ A previous multicenter ED study^15^ had similar findings, reporting that 15.8% of female adolescent patients had sex without contraception in the prior year. Our findings differ from this existing literature and suggest that the rate of contraception use among adolescents in the ED is lower than that of the general adolescent population.

Among study participants who were using contraception, 65.3% reported using a highly effective method, defined as any hormonal method or IUD.^16^ However, LARCs—the most effective option—were the least utilized option among participants at 15.4%. In addition, fewer than one-half (42.7%) of all participants reported male condom use, which has implications for both pregnancy and sexually transmitted infection risk.

Both insurance status and race and ethnicity were found to be significantly associated with overall contraception and, specifically, LARC use. Adolescents with government insurance had significantly lower odds of any contraception use, specifically LARC use, compared with those with commercial insurance. Health insurance status is an important determinant of access to care; therefore, these results indicate that adolescents with government insurance may not have adequate access to contraceptive services.^17^ Non-Hispanic Black adolescents were also less likely to report overall contraception and LARC use compared with non-Hispanic White patients. Individuals who are non-Hispanic Black disproportionately face barriers to health care access due to structural racism.^18^ These findings are concerning in light of racial disparities in unintended pregnancy in the US, with higher rates among non-Hispanic Black and Hispanic youth compared with non-Hispanic White youth.^18^ Thus, expanding ED care to also include sexual health provision may help improve contraception access to marginalized populations.

The overall PRI for our study was 7.89, which translates to an annual pregnancy risk of 7.89% for the study population. The indices of all 6 study sites were higher than the national PRI of 5.0, but lower than a 2017 single-site study conducted among a similar population, which demonstrated a PRI of 9.6.^3^ This indicates a potential downward trend in pregnancy risk among adolescents in the ED, but still higher than the general adolescent population.

Although 10.2% of study participants were potentially eligible to receive EC according to their survey responses, the combined rate of administration among all study sites was remarkably low, with just 5.6% of those eligible receiving EC. Practitioners were not notified about patient survey answers; therefore, determination of EC eligibility was made on the basis of independent sexual history obtained by the practitioner. These results indicated an area for improvement regarding confidential assessment of contraception use and pregnancy risk among patients presenting to pediatric EDs. Previous studies indicate that decreased knowledge, concern for lack of follow-up, time constraints, and lack clinical resources are factors associated with nonprescription of EC by ED practitioners.^19^ After the recent US Supreme Court decision in *Dobbs vs Jackson Women’s Health Organization* (2022), efforts to remove such barriers and improve access to EC for adolescents in the ED will be essential in helping this marginalized patient population maintain a degree of reproductive autonomy.

### Limitations

There are some potential limitations of this study that warrant mentioning. The survey was provided only in English, in accordance with on prior data across the participating study sites. Study participants completed a self-reported survey, which may have been subject to social desirability bias. However, attempts to minimize such bias were made through administration of a confidential, tablet-based questionnaire. Although the literature suggests that the majority of adolescent pregnancies are unintended, our survey did not specifically assess participants’ pregnancy intentions, which may have impacted their contraceptive choices.^2^ Furthermore, the survey was administered exclusively across pediatric EDs affiliated with large tertiary care children’s hospitals, which may not be generalizable to general EDs.

## Conclusions

In conclusion, expanding contraceptive services, including EC, in pediatric EDs may improve access to care and decrease the risk of unintended pregnancy for adolescents. Future studies should focus on strategies to improve EC administration in the ED and explore provision of additional contraception care in the ED.

## Notes

Supplement.
Data Sharing Statement
