# Supplementary material for: Contraception Use and Pregnancy Risk Among Adolescents in Pediatric Emergency Departments
Source: JAMA Netw Open. 2024 Jun 28;7(6):e2418213. doi: 10.1001/jamanetworkopen.2024.18213 (PMC11214110; doi:10.1001/jamanetworkopen.2024.18213)
Supplement: Supplement. — Data Sharing Statement [file jamanetwopen-e2418213-s001.pdf]

Data Sharing Statement

Canter. Contraception Use and Pregnancy Risk Among Adolescents in Pediatric Emergency Departments. *JAMA Netw Open*. Published June 28, 2024.
doi:10.1001/jamanetworkopen.2024.18213

Data
Data available: No

Additional Information
Explanation for why data not available: The data collected represent electronic health record data and sensitive patient data. We did not specify that data would/could be shared with others for such purposes when obtaining informed consent.
